# The exploitation of biofilm by migrant western sandpipers (*Calidrismauri*)

**DOI:** 10.1016/j.heliyon.2023.e17268

**Published:** 2023-06-20

**Authors:** Rachel Canham, James Rourke, Ronald C. Ydenberg

**Affiliations:** a Department of Biological Sciences, Simon Fraser University, Burnaby BC V5A 1S6 Canada; b Canadian Wildlife Service, Environment and Climate Change Canada, Pacific Wildlife Research Centre, 5421 Robertson Road, Delta, British Columbia, V4K3N2, Canada; c Ausenco 18th Floor, 4515 Central Boulevard, Burnaby, British Columbia, V5H 0C6, Canada

**Keywords:** Shorebird, Migration, Grazing

## Abstract

Assessing the quality of migratory shorebird stopover sites requires good measures of food availability. We developed simple methods to measure biofilm grazing by migrant western sandpipers (*Calidris mauri*), a species for which biofilm is an important dietary component. We used a field-portable chlorofluorometer to measure the density of chlorophyll-*a* (Chl-*a*) in surficial biofilms on Roberts Bank, a large intertidal mudflat in British Columbia, Canada, during northward migration.

Chl-*a* density begins at a low level during each diurnal emersion period, and increases steadily during emersion at 4.1 mg m^−2^ h^−1^ for a total of ∼24.6 mg m^−2^ over a typical 6 h emersion period and ∼41 mg m^−2^ over a 10 h emersion period. Western sandpipers grazed at 1.35–1.45 mg Chl-*a* m^−2^ min^−1^, thus biofilm production supports 17.6 min m^−2^ of grazing time during a 6 h low tide period and 29.3 min m^−2^ during a 10 h period. During peak northward migration, the average grazing intensity of western sandpipers over an intertidal emersion period was 3.3–6.4 min m^−2^, suggesting that biofilm accumulation was 2.7–8.8 fold greater than the amount consumed. We found Chl-*a* density was highest (∼65 mg per m^2^) within 40 *m* of the shoreline. Grazing intensity was lowest close to shore, where predation risk from falcon attacks is highest. Grazing intensity peaked at 240 *m* and then declined, lowering Chl-*a* density at greater distances to a uniform level of ∼54 mg m^−2^. These results indicate that interactions between biofilm production and sandpiper grazing underlie spatio-temporal patterns in biofilm abundance on Roberts Bank.

## 1 Introduction

Temperate estuarine mudflats are highly productive ecosystems [1]. They are important for long-distance migrant shorebirds [5,19,38,43,47,49], who forage at these sites to build fuel stores [17,23,37,60]. As a result, many mudflats have high conservation priority, and good measures of food availability at stopover sites are therefore essential in assessing habitat quality.

Until recently the diet of small sandpipers during migration was believed to be composed almost entirely of benthic infauna [63,67]. However, it has since been established that some (e.g. western sandpipers (*Calidris mauri*)) ‘graze’ intertidal biofilm [18,34,35,41], a thin layer (up to a few millimeters thick) at the sediment-water interface consisting of microorganisms, detritus, and sediment [2,65,70] suspended in a matrix of polymers. Biofilms dominate the primary production of turbid, temperate estuaries [33] and are a food source for many invertebrates [24]. Sandpipers graze biofilm using rapid pecking-like motions and a modified tongue which they dab onto the mudflat surface. Foraging by shorebirds has been extensively investigated [51], but due to its recent discovery comparatively little is known about biofilm grazing.

‘Transient epibenthic’ biofilms occur on intertidal mudflat surfaces when ‘epipelic’ microrganisms, predominantly diatoms, migrate to the sediment surface to photosynthesize during daytime low tides [25,26,44,71], often becoming visible to human eyes as brown or golden-brown stains. Biofilm production is reportedly affected by nutrient availability [31], salinity [59], temperature [55], light [56,64], and by the grazing intensity of consumers (e.g. the snail *Peringia ulvae* in Ref. [50]), all of which fluctuate on tidal, daily or seasonal scales.

**Fig. 1:**
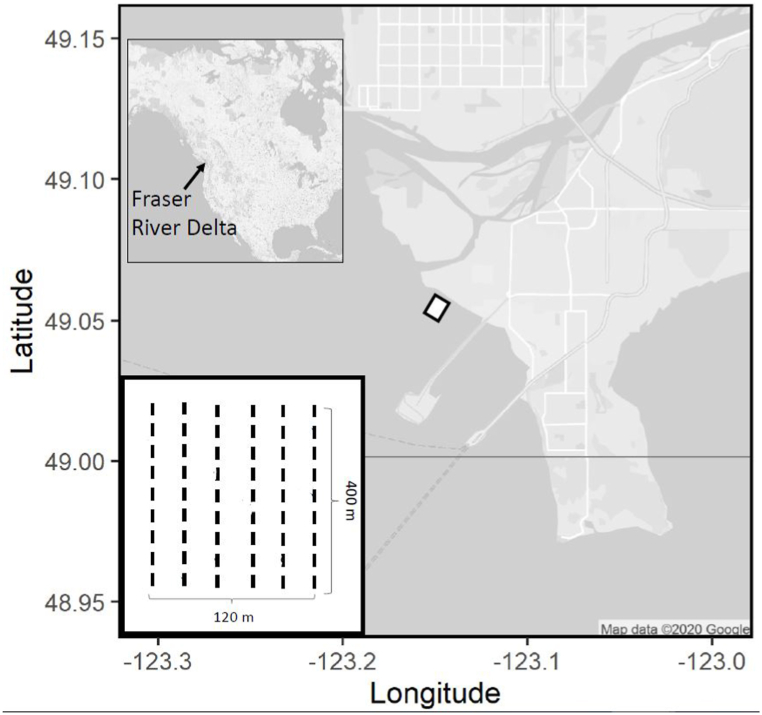
Roberts Bank, Fraser River delta, British Columbia, Canada. Figure adapted from Ref. [9]. The white box defines the study area, located in the dense biofilm zone of the upper intertidal. In 2016 we measured along two 200 *m* transects 25 *m* apart that ran parallel to and ∼100 *m* from the shoreline (design not shown). The inset figure shows the 2017 study design, in which we measured along six parallel, 400 *m* transects, oriented perpendicularly to the shoreline, with the near-shore end located 20 *m* from the vegetation zone. We made this design alteration to capture strong spatial variation across the mudflat noted in 2016.

The biofilm community at Roberts Bank, a mudflat in south-western British Columbia, Canada (49° 03′ 25.20″ N, 123° 10′ 23.40″ W; Fig. 1) is a typical mixed, estuarine community dominated by *Nitzschia*, *Navicula*, and *Achnanthidium* diatom genera, common on estuaries throughout the world [3,69,72]. Biofilm grows over the entire bank, but its density is highest on muddy areas of the upper intertidal, at ∼3.2 *m* datum and higher. The sediment below the surface layer here is anoxic, and contains few invertebrates that might also consume biofilm.

Shorebirds make use of Roberts Bank throughout much of the year. Pacific dunlins (*Calidris alpina pacifica*) are winter residents at Roberts Bank and western sandpipers stopover during north and southbound migration along the Pacific flyway, between tropical, non-breeding and Arctic, breeding areas. However, the western sandpiper diet contains a substantial portion of biofilm [35], which forms only a small component of the Pacific dunlin diet [66]. Western sandpipers forage in flocks, arriving as the high tide recedes and spreading out over the mudflat. Individuals and small groups walk and run across large portions of the mudflat, or fly between foraging aggregations [28]. Northbound migration takes place mid-April to early May, with 10,000–50,000 shorebirds present most days, rising to a peak in the last days of April [10,14]. The length-of-stay of individual birds at Roberts Bank is only a few days (1.8–3.2 d northbound; 3.8–9.6 d southbound; [15]. The southbound migration period is more protracted (July and August), with no strong seasonal peak in numbers. Shorebirds also use other sites throughout the region not used during northward migration [7,27,40]. As a result, many fewer western sandpipers are present each day on Roberts Bank during southbound migration.

Chemical extraction methods are used to assess the amount and constituents of biofilm in samples collected from mudflat surfaces (e.g. Refs. [12,45,46,48]). These are labour-intensive, time-consuming and destructive, and hence preclude rapid, repeated measures on a small scale. Reflectance measurements (e.g. NDVI [32]); can be used to study large- and mesoscale spatial distribution patterns in Chl-*a* density [32] but require an airborne or satellite platform, thus measures repeated at short intervals are generally not possible.

Our aim in this study was to measure biofilm abundance on Roberts Bank on temporal and spatial scales relevant to foraging by sandpipers. We used a field-portable chlorofluorometer to make low-cost, rapid, repeated, and non-destructive *in situ* measurements to estimate the rate with which biofilm accumulates on the mudflat and the rate at which it is removed by grazing western sandpipers.

## 2 Methods

### 2.1 Chlorofluorometer measures

Chlorophyll fluorescence [42] is widely used in botany and ecophysiological studies, including of marine and microalgae [4], and has previously been used to measure the Chl-a concentration of biofilm in both laboratory (in surficial sediments transported to the lab [61]); and field [33,50] studies. We used a field-portable chlorofluorometer (Opti-science model CCM-300) to make Chl-*a* density measurements on Roberts Bank during western sandpiper northward migration during two years. Chl-*a* density correlates well with that of biofilm components considered nutritious for sandpipers such as carbohydrates and, especially, fatty acids. Use of the instrument, calibration and its validation for these measures are described in detail in the Appendix.

The study area and sampling transect locations are shown in Fig. 1. In 2016 (April 23–29) two transects situated 25 *m* apart and 200 *m* in length, were delineated in the dense biofilm zone of the upper intertidal parallel to and ∼200 *m* from the shoreline. In 2017 we measured along six parallel, 400 *m* transects, oriented perpendicularly to the shoreline, with the near-shore end located 20 *m* from the vegetation zone. We altered transect orientation to assess the decline in biofilm density (noted in 2016) with distance from shore. Measures were made on 13 days between 20 April and 7 May, 2017, during the shorebird northward (high-intensity grazing) migration period, and on 10 days (weekly intervals, July and August) during the southward (low-intensity grazing) migration period. Measurements began 2–3 h after the high tide, and required 3–4 h to complete. The order and direction with which we measured each transect was randomized. After each Chl-*a* density measurement was recorded, we estimated a corresponding measure of sandpiper grazing intensity using the procedure described below.

We placed nine exclosures across the study area in 2017. Four white (PVC) 30 cm stakes marked the corners of a 1 m^2^ exclosure plot. We tied flagging tape around the perimeter and across the interior, with 15 cm streamers on each stake. As judged by the absence of footprints (easily visible in the soft mudflat surface; see photo in Ref. [9]) these exclosures were effective at excluding western sandpipers. We measured Chl-*a* density inside each exclosure and on an adjacent control plot on each of six days during northward migration.

### 2.2 Grazing intensity

We measured western sandpiper grazing intensity using the procedure developed by Ref. [52]. Western sandpipers defecate distinctive white circular droppings ∼1.5 cm in diameter once every 2.2 min that are easily seen on the mudflat surface [9]. Therefore, droppings provide a sensitive indicator of accumulated grazing time prior to the time of measurement [9]. Droppings are washed away at high tide, so there is no accumulation from previous days [67]. The dropping count thus represents the grazing time accumulated since the previous high tide. We averaged the dropping density in five 1.0 m^2^ quadrats randomly placed within a 5 *m* radius of each chlorofluorometer measurement station, expressing grazing intensity as droppings m^−2^.

We calculated emersion duration as the interval between the time of measurement and the time at which the receding tide reached a height of 3.2 *m*, based on tidal predictions for the nearby Tsawwassen tidal station [21]. Daily daytime mudflat emersion was determined by cross-referencing tide height predictions [21] above 3.2 *m* with time of sunrise and sunset [22] for Tsawwassen, BC, from April 20 to May 7, 2017.

### 2.3 Western sandpiper abundance

Environment and Climate Change Canada (ECCC, a federal government service) conducted daily shorebird surveys along the entire shoreline length of Roberts Bank throughout each spring migration period [14]. We used these data as a measure of western sandpiper abundance on the days we measured biofilm.

### 2.4 Data analysis

We made several comparisons to assess the impact of shorebird grazing on biofilm density. First, from the transects established in 2016 we obtained 144 paired measures of biofilm density and grazing intensity, spread throughout the emersion period. Each of the 144 stations was classified as ‘ungrazed’ (if the dropping density was zero; n = 86), or ‘grazed’ (dropping density >0; n = 58). The Chl-*a* measures were compared between these groups using Welch's unequal variances *t*-test (one-tailed). Assumptions of equal variance and normal distributions were both met.

Second, using these data we estimated the rate of biofilm accumulation using a linear mixed effects model to assess Chl-*a* density in relation to the fixed effects of mudflat emersion duration (i.e. time since tidal exposure; h) and dropping density. We included sampling date as a random effect.

Third, we analyzed and compared the density of Chl-*a* during the northward (high grazing intensity; N = 898) and southward (low grazing intensity; N = 703) migrations using separate linear mixed effects models. The models analyzed Chl*-a* density in relation to distance from shore and dropping density, with sampling date and transect identity as random effects. Of the total 2251 measures, 650 (29%) were ‘non-detects’. Using logistic regression, we found no relationships between the incidence of non-detects and dropping density or any of the following variables: distance along transect, transect ID, and northward vs. southward migration. We therefore treated these as ‘Missing Completely at Random’ [36] in analyses.

Lastly, we compared Chl*-a* densities in each of the nine exclosures with corresponding control plots on the six measurement days in 2017.

## 3 Results

The number of western sandpipers surveyed at Roberts Bank differed between the northward and southward migration periods as documented in previous years (Table 1), with the daily number higher during northward migration by a factor of ∼70 (2017) – 545 (2016). During northward migration, averages of 32,188 (2016) and 13,918 (2017) sandpipers were surveyed on the days coinciding with the field measurements made in our study. In contrast, during southward migration daily averages of only 59 (2016) and 200 (2017) sandpipers were tallied (ECCC, unpublished).

**Table 1:** Mean daily western sandpiper counts at Roberts Bank during northward (spring) and southward (summer) migration. Methods are described in Ref. [14]. Northward migration counts are made daily by Environment and Climate Change Canada); N is the number of census days corresponding with days on which we measured biofilm. Southward migration counts provided by Ref. [27].

Migration	Year	N	Mean	SD
Northward	2016	6	32,188	13,599
	2017	15	13,918	11,284
Southward	2016	4	59	54
	2017	5	200	116

### 3.1 Grazing intensity

The mean dropping density measured during northward migration was 1.5 droppings m^−2^ in 2016 (N = 144) and 2.9 droppings m^−2^ in 2017 (N = 898), representing grazing intensities of 3.3–6.4 grazing minutes m^−2^. We detected no droppings in 703 samples during the 2017 southward migration. Note that with 70–545 fold fewer western sandpipers, the expected dropping density is only ∼0.041–0.003 m^-2^, low enough that droppings may not be detected, even in a sample of 703.

### 3.2 Biofilm increase rate

**Fig. 2:**
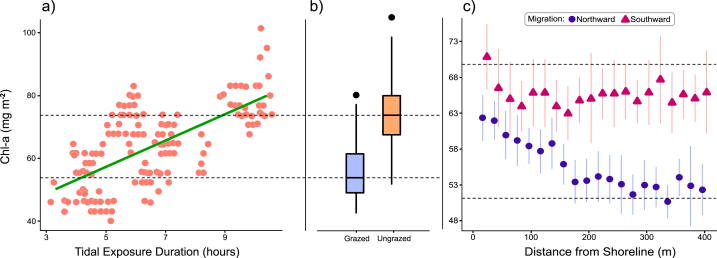
Biofilm growth, and removal by grazing. The dashed horizontal lines are set to the medians of the box plots in panel b to aid in visual comparisons. a) Biofilm density (mg Chl-*a* m^−2^) rises linearly during tidal emersion periods at 4.1 mg m^−2^ h^−1^. Each point (N = 144) represents a measure along the 2016 transects. b) Biofilm density is lower by 11.9 mg m^−2^ on stations grazed by western sandpipers than on ungrazed stations. Measures made in 2016. Box plots show the median (horizontal bar), the 25% and 75% quartiles (upper and lower edges of the box), and the range (vertical lines) excluding outliers (dots). c) The biofilm profile with distance from shore differs between northward (high grazing intensity - solid dots) and southward (low grazing intensity - triangles) migration periods. Points represent the average of measures made on 10 days in July and August of 2017. Bars are standard errors.

Chl*-a* density increased with emersion time, rising linearly at 4.1 (±95% CI 1.17) mg m^−2^ h^−1^ during diurnal emersion (F_1,143_, = 55.3p < 0.001; Fig. 2a). When only grazed stations are considered, the rate of increase is 3.9 (±95% CI 1.4) mg m^−2^ h^−1^ (F_1, 57_ = 35.0, p < 0.001). When only ungrazed stations are considered, the rate of increase is 4.6 (±95% CI 1.96) mg m^−2^ h^−1^ (F_1,85_ = 30.0, p < 0.001). These rates do not differ significantly (Welch's unequal variances *t*-test, t = 0.591, p = 0.555).

During the 2017 study period, daily daytime emersion ranged from ∼7 h–14 h and averaged ∼10 h^−1^ day (±2.5 SD). Emersion duration during Chl-*a* density sampling ranged from ∼3 h–12 h and averaged ∼6 h since tidal exposure.

### 3.3 Impact of grazing on biofilm density

We estimated the impact of grazing on biofilm density using three comparisons (Table 2). First, we compared Chl-*a* density between grazed and ungrazed stations measured in spring 2016. The overall mean Chl-*a* density at ungrazed stations was 66.6 mg m^−2^ (N = 86, ±95% CI 1.76), while the mean density at grazed stations was 54.6 mg m^−2^ (N = 58, 95% ± CI 1.37), lower by 11.9 mg m^−2^ (±95% CI 2.35; t = 7.92, p-value <0.001, Table 2; Fig. 2b). Grazing intensity at grazed stations was on average 4.0 (±95% CI 0.78) droppings m^−2^, while the dropping density at ungrazed stations was (by definition) 0.

**Table 2:** Three comparisons of Chl-*a* density at Roberts Bank between stations ‘grazed’ and ‘ungrazed’ by western sandpipers: (1) grazed and ungrazed stations in 2016; (2) northward (grazed) and southward (ungrazed) migration in 2017; (3) control (grazed) and exclosure (ungrazed) stations in 2017. For each category we report the mean Chl-*a* density (mean, in mg m^−2^), sample size (N), standard error of the mean (SE), the 95% confidence interval, and the mean dropping density (m^−2^). The column labelled ‘difference’ gives the difference between the estimates, the value of the statistic used for comparison (detailed in Methods) and the probability level.

Comparison	N	Chl-*a*	SE	95% CI	droppings	difference, in mg m^−2^ (mg m^−2^) (no. m^−2^) (statistic, probability)
(1) grazed	58	54.6	0.7	52.8–56.6	4.0	11.9
ungrazed	86	66.6	0.9	65.1–68.0	0.0	(t = 7.92, p < 0.001)
(2) northward	898	56.2	0.3	55.8–56.8	2.9	9.3
southward	86	66.6	0.9	65.1–68.0	0.0	(F = 210, p < 0.001)
(3) control	9	49.1	1.7		0.3	9.3
exclosure	9	54.4	1.6		0.0	(t = 3.00, p = 0.05)

Second, we compared Chl-*a* density measures between northward (high grazing intensity) and southward (low grazing intensity) migration periods in 2017. The mean Chl-*a* density during southward migration was 65.5 mg Chl-*a* m^−2^ (10 days, N = 703, ±95% CI 0.59), while during northward migration the overall density was 56.2 mg Chl-*a* m^−2^ (13 days, N = 898 ± 95% CI 1.96), lower by 9.3 mg m^−2^ (F_1,1571_ = 210, p < 0.001; Fig. 2c; Table 2). Confirming the difference in grazing intensity, the average dropping density during northward migration was 2.9 droppings m^−2^, and no droppings were detected during southward migration.

Third, the nine exclosures and their adjacent control plots were measured on each of 6 days in 2017 during northward migration. Chl*-a* density inside exclosures was 5.3 mg m^−2^ (54.4 mg m^−2^; n = 54, ±95% CI 3.33) higher than the adjacent control plots (49.1 mg m^−2^; n = 54, ±95% CI 3.14) and was only marginally significant (t = 3.00, p = 0.05). Average dropping density in the control plots was 0.3 (±95% CI 0.18) droppings m^−2^.

### 3.4 Spatial patterns

Fig. 2c reveals a strong difference in the spatial pattern of Chl-*a* density across the mudflat between the north- and southward migration periods. During southward migration, overall density was 65.5 mg Chl-*a* m^−2^, and we detected no decline in Chl-*a* density with distance from shore (‘ungrazed’; F_19, 900_ = 0.4; p = 0.531). In strong contrast, Chl*-a* density fell steadily with distance from shore during the northward migration period, decreasing by 0.03 (±95% CI 0.004) mg m^−2^ for every 1 *m* increase in distance (holding dropping density constant; F = 181; p < 0.001), and by 0.6 (±95% CI 0.19) mg m^−2^ with each additional dropping (F_19, 1311_ = 9.05, p = 0.003). The density of Chl-*a* was greater at any distance from the shoreline during southward than northward migration, with the difference increasing from near zero at the shoreline, to about 11 mg m^−2^ at ∼240–400 *m* from shore.

**Fig. 3:**
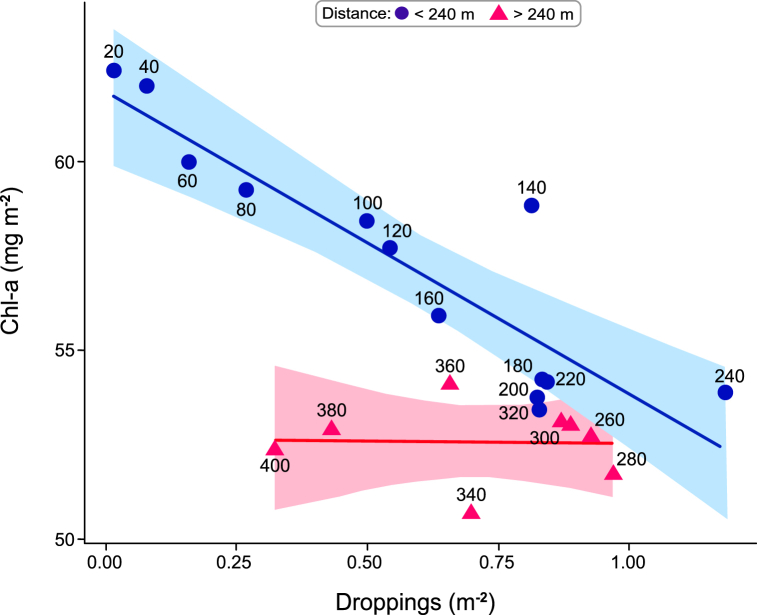
Mean dropping (m^−2^; x axis) and biofilm (mg Chl-*a* m^−2^; y-axis) densities in the upper intertidal of Roberts Bank during 2017 western sandpiper northward migration. The lines are best fit regressions (shading is SE), divided into sections 20–240 m (−7.9 mg m^−2^; R^2^ = 0.8, p < 0.001) and 260–400 m (−0.24 mg m^−2^, R^2^ = 0.003, p = 0.89). The distance of each point is given. 20–240 *m*: biofilm density falls from 62 to ∼53 mg m^−2^, and dropping density rises. 260–400 *m*: biofilm density is constant at 53 mg m^−2^ while dropping density falls.

Dropping density also revealed a distinct spatial pattern, summarized in Fig. 3. Dropping density is very low at the shoreline, and rose to a peak at a distance of 240 *m* from shore. Chl*-a* density fell by 7.9 mg m^−2^ (±95% CI 2.55; R^2^ = 0.8, p < 0.001) over the same distance. From 240 *m* from the shoreline to the 400 *m* extent of the transect, Chl-*a* density was constant at an average density of ∼52.7 mg m^−2^ (±95% CI 3.33; R^2^ = 0.003, p = 0.89), while dropping density fell with distance.

## 4 Discussion

Our results provide some of the first *in situ* field measurements of the rates and short-term dynamics of biofilm growth and grazing by western sandpipers. The density of biofilm on the Roberts Bank upper intertidal study site is lowest just after tidal emersion, climbs linearly at a rate of 4.1 mg m^−2^ per hour, reaching ∼50 mg m^−2^ 3 h after emersion, and ∼62 mg m^−2^ after 6 h. Both the vertical (‘epipelic’) migration of diatoms [26,44] and reproduction may contribute to this increase, though we are as yet unable to assess their relative contributions. The increase estimate of 4.1 mg m^−2^ h^−1^ is based on the pooled ‘grazed’ and ‘ungrazed’ stations because the separate growth rate estimates of 3.9 and 4.6 mg m^−2^ h^−1^, respectively, do not differ statistically. However, the time at which stations had been grazed prior to the Chl-*a* measurement is unknown, and could not be controlled for in this analysis. This remains an unresolved point.

Previous studies by Refs. [18,34,35] established that western sandpiper bill and tongue morphology is modified for the consumption of biofilm, and that biofilm is an important dietary component. These studies also provide a behavioral measure of the biofilm grazing rate (121 foraging actions min^−1^), and estimated that each foraging action obtains 2.6 mg of biofilm (wet weight). This equates to a foraging rate of 1.6 mg Chl-*a* grazing min^−1^ (assuming that Chl*-a* content is 2.5% of the dry weight, and that the dry weight is 20% of the wet weight).

We derived similar estimates of western sandpiper grazing that measurably reduced Chl-*a* density on the mudflat. During 2016 northward migration, the difference between grazed and ungrazed stations was 11.9 mg Chl*-a* m^−2^. Grazed stations had an average dropping density of 4.0 m^-2^, which at 2.2 min per dropping [9] represents 8.8 min of grazing time. Combining these measures (8.8 grazing min m^−2^ lowers Chl*-a* density by 11.9 mg m^−2^) suggests a grazing rate of 1.35 mg Chl*-a* m^−2^ min^−1^. In 2017, the average biofilm density during northward migration was 56.2 mg Chl-*a* m^−2^, lower by 9.3 mg m^−2^ than the southward average density of 65.5 mg Chl-*a* m^−2^. The average dropping density during northward migration was 2.9 m^-2^, which represents 6.4 min of grazing time. Combining these measures (6.4 min of grazing lowered Chl-*a* density by 9.3 mg m^−2^) estimates a grazing rate of 1.45 mg Chl-a m^−2^ min^−1^. Though based on different comparisons in two separate years, these independent estimates match closely.

The rate of Chl-*a* increase of 4.1 mg m^−2^ h^−1^ is therefore estimated to be able to sustain 2.9 min of western sandpiper grazing m^−2^ h^−1^, for a total of 17.5 grazing min over a typical 6 h intertidal emersion period, and 29.3 grazing minutes over a 10 h period. This represents 7.9 and 13.3 droppings m^−2^, respectively, (@ 2.2 grazing minutes per dropping), which exceeds by a wide margin the average dropping densities measured over the entire study area during 2016 and 2017. These estimates suggest that the accumulation of biofilm throughout a 6 h emersion period exceeds removal by a factor of 2.7 (2017 dropping density 2.9 m^-2^) to 5.3 (2016 dropping density 1.5 m^-2^), and a factor of 4.6–8.8 during a 10 h emersion period. Derived over two years of study, these estimates, apply to the biofilm-dense areas of the upper intertidal at Roberts Bank, where, based on estuary-wide surveys (Hemmera, unpubl. data) the highest dropping densities on the entire estuary occur. We assume that the grazing intensity, and biofilm accumulation rate, is lower in other regions of the mudflat.

Estimating the grazing rate based on the difference between the exclosures and their control quadrats yielded a different value. Analogous comparisons to those made above yield a grazing rate estimate of 8.0 mg Chl-*a* m^−2^ grazing min^−1^ – almost six times higher than those above. There are reasons to be skeptical of this estimate. The Chl-*a* density in the exclosures as well as the control quadrats was lower than that measured elsewhere in either 2016 or 2017, even though the exclosures had not been grazed. Exclosures often create inadvertent effects, including alteration of air or water flow, shading, extra heating, sediment deposition, or alterations in biological and chemical aspects of the environment [57,62,67]. Frequently, we found seaweed and other marine debris caught on the exclosure stakes, which may have blocked sunlight or water flow. Such debris was removed, but doing so this may also have removed some of the biofilm. We conclude that the grazing rate estimate based on the exclosure comparisons is unreliable.

The results of our study reveal a dominant temporal pattern in biofilm abundance on the mudflat; the tidal rhythm (summarized in Fig. 2). At the start of tidal emersion, biofilm surface density is at a reduced level and accumulates steadily to the same peak density (Fig. 2a), even when western sandpiper grazing intensity is low during the summer. During tidal immersion, biofilm surface density is reduced to the previous day's starting level, due perhaps to invertebrate consumption, dispersal, removal by other processes, or by reverse migration of diatoms. This process of biofilm reduction and increase is repeated with each tidal cycle. Although biofilm density is lower in February than in April and May [58], the daily peak in mid-April reached the same level measured in June, July and August. The reason is perhaps that the daily duration of diurnal emersion increases greatly around the spring equinox, as daylength rapidly increases and the tidal pattern shifts from primarily nocturnal to primarily diurnal emersion periods.

The strongest spatial pattern in Chl-*a* density was the decline with distance from shore observed during northward migration (Fig. 2c), a pattern absent during southward migration. Overall, Chl-*a* density was higher during southward migration, though note that differences close to shore are slight. Seasonal climatic factors could perhaps account for this difference. Alternatively, or in addition, the onshore sediment grain-size distribution with finer particles close to shore [30], tidal patterns, or nutrient input could create a spatial gradient of biofilm production. However, for these factors to explain the absence of this spatial gradient during southward migration requires that they operate during spring, but not in summer. This seems unlikely.

The spatial pattern we observed could be attributable to the variation in grazing intensity if, as in other grazing systems, top-down processes are as important as bottom-up processes in determining the standing crop [11,54,56]. Sandpipers forage less close to the shore [8,53] where predation risk is higher, because vegetation and other visual obstacles afford falcons opportunities for concealment and surprise attack [13]. On Roberts Bank biofilm is grazed down to about 54 mg m^−2^ over much of the upper intertidal zone (Fig. 2c), but within 240 *m* of shore its density rises with increasing proximity to the shoreline. This gradient can be interpreted as a form of giving-up density (GUD), in which the amount of food foragers leave behind is a measure of the danger they perceive is present at that site [6].

## 5 Conclusion

We conclude that Chl-*a* density measures derived from a field-portable chlorofluorometer provide a reliable and useful way to assess the abundance of biofilm in a manner relevant to foraging by western sandpipers. Our measurements indicate that biofilm production on Roberts Bank during a tidal emersion period is cyclical and exceeds the amount grazed by sandpipers by 2.7–8.8 times. Western sandpiper grazing was much higher during northward than southward migration, and was highest in an area 240–400 *m* from shore where the risk of predation was lower. Our results indicate that interactions between biofilm production and sandpiper grazing underlie spatio-temporal patterns in biofilm abundance on an important shorebird stopover site along the Pacific flyway. These findings provide information on biofilm dynamics and shorebird grazing patterns at Roberts Bank, and may support baseline studies and future research into the importance of biofilm for migrating western sandpipers.

## Declaration of competing interest

The authors declare that they have no known competing financial interests or personal relationships that could have appeared to influence the work reported in this paper.

## Figures and Tables

**Fig. A1:**
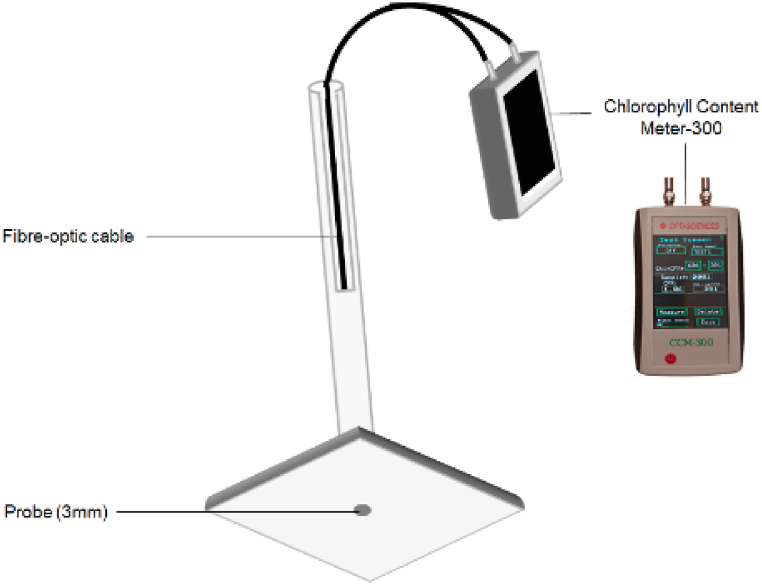
The CCM-300 unit (12 cm × 9 cm x 3 cm) (a) was secured to a stand composed of an arm (60 cm length) and a square base (17 cm × 17 cm) with a 3.1 mm hole drilled through its center. To make a measurement, the stand is placed on the mud, and using the CCM-300's built-in function a series of five repeat measurements made, of which the average is logged automatically along with the time of measurement. The probe is rinsed with clean water after each set of measures. The instrument works reliably, even under very wet and muddy conditions.

**Fig. A2:**
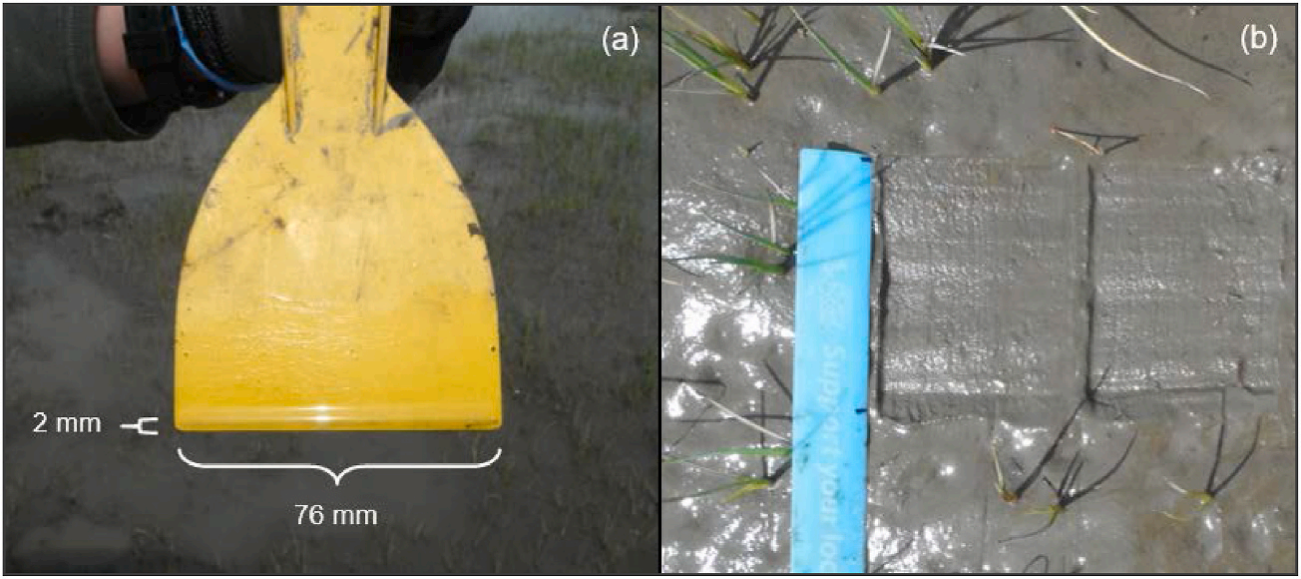
Plastic putty knife (with dimensions) used to scrape biofilm samples (a), and a photo showing the mudflat surface after the sample was collected. The ruler indicates the 10 cm scrape (b).

**Fig. A3:**
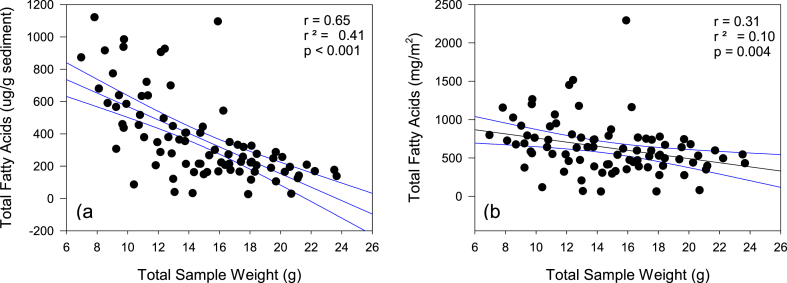
Relationships between total fatty acid content and the amount of sediment in samples, expressed as (a) the concentration (micrograms per gram of dry sediment); and (b) abundance per unit area (mg/m^2^), based on 84 samples collected April 13 to May 17, 2018.

**Table A1:** Calibration equations describing the conversion of ‘CFR’ (chlorophyll fluorescence ratio) readings of the CCM-300 (see https://www.optisci.com/ccm-300.html) into Chl-*a* density (units mg m^−2^). ‘Gitelson’ is the equation originally developed by Ref. [20]; based on elm, oak and vine leaves, and is provided here for comparison. ‘2017’ is the calibration obtained 2017, and ‘2021’ that in 2021. The two do not differ statistically. ‘Pooled’ is the calibration equation based on these samples pooled together.

Year	Calibration equation		n	r	p
	Intercept	Slope			
2017	55	307	7	0.75	<0.025
2021	59	301	14	0.54	<0.025
Pooled	51	385	21	0.54	<0.01
Gitelson	391	634	55	0.98	<0.001

**Table A2:** Summary of correlations between the density of Chl-*a* (mg m^−2^) and the level of SFA (= saturated fatty acids), MUFA (= monosaturated fatty acids), PUFA (= polysaturated fatty acids), all fatty acids, and total carbohydrates (all in mg m^−2^). Significance levels are Bonferonni-corrected for the multiple comparisons made in the full analysis carried out in each of the three years.

Year	2016	2017	2018
No. Sampling sessions	6	17	12
No. Samples @ 7 per session	42	119	84
**Correlation statistics (r/significance level) between Chl-a and:**			
SFA	0.56/ = 0.084	0.49/<0.05	0.53/<0.001
MUFA	0.53/ = 0.213	0.53/<0.001	0.52/<0.001
PUFA	0.66/ = 0.002	0.52/<0.001	0.61/<0.001
All fatty acids	0.59/ = 0.033	0.57/<0.001	0.59/<0.001
Total carbohydrates	0.79/<0.001	0.43/n.s.	0.57/<0.001

## APPENDIX

### Background

This Appendix provides details on use of the CCM-300 to predict the levels of Chl-*a* and other components of biofilm. We describe field measurement methods, the collection of sediment samples of biofilm, procedures for the laboratory analysis of components of these samples, and the calibration method.

### Field chlorofluorometer measures

Chlorophyll fluorescence [42] is widely used in botany and ecophysiological studies, including of marine and microalgae [4]. It has been used to measure the Chl-*a* concentration of biofilm in both laboratory (in surficial sediments transported to the lab [61]; and field [33,50] studies. We used a field-portable chlorofluorometer (Opti-science model CCM-300) designed to measure the Chl-*a* content of small, curved or uneven plant tissue specimens. This instrument was validated by Ref. [20]; who found that the ‘chlorophyll fluorescence ratio’ (CFR; the ratio of the wavelengths) is linearly proportional to the Chl-*a* content over a large range (41–675 mg m^−2^). It has since been used successfully in a wide variety of applications including tiny seedlings, conifer needles, chlorolichens, moss, grasses, cacti, corals, fruit peels and others (https://www.optisci.com/ccm-300.html gives a full list).

We mounted the CCM-300 on a portable stand with a wide base (Figure A1) enabling it to rest lightly and steadily on the mudflat surface. The fibre-optic cable was threaded through the hollow arm of the stand, with the small (3 mm) cylindrical probe snugly fitted into a hole in the base, slightly recessed (0.5 mm) from the mudflat surface. Measurement at a ‘station’ was composed of 5 successive readings of the CFR ratio, each requiring up to 10s, moving the stand a few centimeters between each. Each reading was logged automatically, along with the date and time, and the average later computed. Measurements were made during the northward passage period of western sandpipers, beginning 2–3 h after high tide, at ∼20 *m* intervals (stations approximated by pacing) along transects. The soft mudflat surface made walking slow and a visible trackway quickly developed, so we were careful to restrict our movements to narrow well-defined paths, keeping the mudflat along both sides of each pathway completely free of tracks.

### Biofilm and sediment samples

Biofilm samples were obtained at seven stations in the upper intertidal of Roberts Bank, pre-selected to give a wide range in biofilm density. We used handheld GPS units to locate the stations while in the field. Sampling was initiated approximately ∼1.5 h after emersion on a falling tide, providing enough time for biofilm to migrate to the surface [26]. The order with which stations were sampled varied between sample days. At each station two replicate plots were randomly located 5–25 *m* from the station center (7 stations × 2 replicates/station = 14 sampling plots daily), marked using pin-flags, and sampled again on subsequent surveys. Surveys were conducted only on days with no precipitation.

#### Sample Collection

Biofilm sediment samples were collected using a 7.6 cm wide putty knife to carefully scrape the top 2 mm from a 10 cm long strip of biofilm (area = 76 cm^2^; Figure A2), using the 2 mm angled edge of the scraper to control the depth of the scrape as carefully as possible. The mudflat surface is not perfectly smooth, varies from watery to sticky, and objects such as vegetation and large particles could disrupt the scrape. Sample weights therefore varied considerably (∼7–35 g dry mass). We consider this further below.

In 2016, separate samples were collected at each sampling plot for laboratory analysis of chlorophyll *a*, and total carbohydrates and fatty acids. In 2017, chlorophyll *a*, carbohydrates, and fatty acids were derived from the same sample collected at each sampling plot. Immediately after collection, each sample was placed in a 60 mL centrifuge tube, labelled, and sealed in an amber bag, also labelled. The samples were placed in a cooler with dry ice, and transferred to a −80 °C freezer at the end of each field day, and later shipped for analysis.

#### Sample Processing

Samples were couriered to Aquatech Enviroscience Laboratories, Inc. (AEL) (North Saanich, B.C.). Transit time to the lab was approximately 6 h. The samples were shipped on dry ice and remained below −60 °C at all times. Laboratory personnel verified the temperature record, and confirmed the samples were accounted for in the Chain of Custody (COC). A unique laboratory ID number was issued to each sample vial, logged, and linked to the field number provided on the centrifuge tube and sealed amber bag to assure tracking of each sample. Each sample was weighed, freeze-dried at −60 °C, and re-weighed to determine its dry weight. The dried sample was ground to fine powder using a pestle and mortar. Sub-samples were drawn for the measurement of Chl-*a* (40–200 mg subsample, depending on organic content), total carbohydrates (25 mg–70 mg subsample), and fatty acid analyses (940–1550 mg).

#### Chlorophyll *a* protocol

Chl-*a* was extracted from the sub-sample with high performance liquid chromatography (HPLC) grade acetone: methanol at a 90:10 ratio. The measurements were performed using a Fluorescent Spectrometer from TURNER (model 7200–000, using Trilogy Module CHL-NA, Model #46, Turner Designs, Sunnyvale, CA, USA). Prior to extraction, samples were re-lyophilised for 2 h to remove any residual water, frost or ice. Chl-*a* was extracted by mixing 50–100 mg of dry sediment in 5 mL of degassed mixture of acetone: methanol (90:10 b y volume) in 15 mL glass tube, then vortexed. The sample tubes were flushed with N_2_, wrapped in aluminium foil to protect from the light, then stored in the dark at −10 to −20 °C for 12 h, before being vortexed again. The tubes were then centrifuged for 5 min to achieve a better separation of the solvent extract and sediment. An aliquot of the extract (1–2 mL) was transferred into a clean 12 × 75 mm disposable glass test tube, where raw fluorescence units (RFU) were read on the fluorimeter.

#### Total carbohydrate protocol

The analytical protocol used for total carbohydrate in sediment samples followed the methodologies described by Refs. [16,68]. The analytical system included a Pharmacia Biotech model Ultraspec 2000 and a UV–Vis Spectrometer.

Total carbohydrate concentrations were measured by creating a phenol-sulphuric acid assay. Two mL of distilled H_2_O was added to 100 mg of freeze-dried homogenised sediment in a 15 mL conical plastic tube. Then, 1 mL of 5% phenol aqueous solution and 5 mL of concentrated H_2_SO_4_ was added to the mixture. Once the production of gas ceased, the mixture was shaken in a vortex and put in a water bath for 1 h at 30 °C. The mixture was then centrifuged at 3000 RPM for 15 min. The tubes were allowed to stand for 10 min, then shaken and placed for 10–20 min in a water bath at 30 °C. The absorbance of the supernatant was measured using a spectrometer at 485 nm.

#### Fatty acid protocol

Sub-samples were lyophilised using a Labconco Freezone 2.5 Freeze Drier. A portion (∼0.5 g) of the homogenised dry sediment sample was used to determine total organic carbon (TOC), needed to determine the sample amount necessary to provide the 10 mg of TOC for FAME analysis. Fatty acids were analyzed as methyl esters using a gas chromatograph (GC; Varian CP-3800; Varian) equipped with a flame ionisation detector (FID) or Ion –Trap Mass Spectrometer (ITMS) [29]. The methyl esters were prepared by *trans*-esterifying the lipid extract in H_2_SO_4_–CH_3_OH at 50 °C for 16 h [29]. The FAMEs were analyzed on a Supelco 2560 Capillary Column (100 *m*, 0.25 mm inner diameter, and 0.2 μm film thickness). Helium was used as the carrier gas (1 mL/min flow rate). The following temperature ramp was used: 65 °C for 0.5 min, holding at 195 °C for 15 min after ramping at 40 °C/minute, and holding at 240 °C for 10 min after ramping at 2 °C/minute. Foe FID detector, helium (make-up gas) and air (combustion) had flow rates of 30 and 300 mL/min, respectively. The sample injector was isothermal at 250 °C. FAMEs were identified by comparison of their retention times with known standards (37-component FAME mix, Supelco 47,885) and corresponding mass spectra using ITMS set in total ion count (TIC) mode. Quantification was performed with reference to seven-point calibration curves derived from 2.5, 50, 100, 250, 500, 1,000, and 2000 ng/uL solutions of the FAME standard.

### Data Treatment and Statistical Analysis

Care was exercised while sampling to remove only the top 2 mm of sediment, but variation in sampling depth over the length of the 10 cm scrape proved unavoidable. Along with variable sediment grain size and water content, the dry mass of samples from 7 to 35 g. The concentration of biofilm components (per unit dry weight of the dried sample) is diluted in heavier samples (Fig. 3A, left panel), because these likely included more deeper sediment, and biofilm is concentrated in the top 2 mm of sediment [26,39]. To control for this effect, each sample's dry weight was multiplied by the concentration of the component in question (Chl-*a*, carbohydrates, and fatty acids, in micrograms per gram of dry sediment) and divided by the area of the scrape (0.0076 m^2^), expressing the density as amount per unit area of intertidal mudflat surface (e.g. mg fatty acids/m^2^; Fig. 3A, right panel). An area-based measure for the density of food is consistent with the literature on shorebird foraging.

### Chlorofluorometer calibration

We developed a calibration equation using correlations between CCM-300 CFR measures and laboratory-determined Chl-*a* content of simultaneously-collected biofilm samples. Calibration was carried out in 2017 and again in 2021. The two equations are virtually identical (Table A1), even though different CCM-300 units were used. This lends considerable confidence that the result is reliable. Each relation is noisier than that reported by Ref. [20] in their validation of this instrument, and the slope is lower. Both these outcomes are expected, because small scale variability in Chl-*a* density is undoubtedly higher in biofilm on surficial sediments than in the deciduous leaves used by Ref. [20]; and the concentration of Chl-*a* in biofilm is lower than in leaf tissue, due to the presence of EPS and sediment. We used the pooled calibration to calculate Chl*-a* density.

#### Nutritive components of biofilm

We assessed correlations between Chl-*a* and the nutritive (fatty acids, carbohydrates) components of biofilm. A total of 245 samples were collected at the seven stations on Roberts Bank, on 35 sampling days between mid-April and mid-May 2016, 2017 and 2018. Results are summarized in Table A2.

Correlations of Chl-*a* with the levels of saturated fatty acids (SFA), monosaturated fatty acids (MUFA), polysaturated fatty acids (PUFA), as well as the total of all fatty acids, are all positive and overwhelmingly significant. Most (52/63) comparisons between Chl-*a* and levels of the 21 individual fatty acids assessed (data not reported here) are also significantly positive. The non-significant comparisons involve individual fatty acids present in very low concentrations. Note that correlation co-efficients retain about the same value over the three years, while significance levels rise. The increase in significance is attributable to reduction of the variance, likely due to improvement in field technique over the three successive years of sampling.

Correlations between Chl-*a* and total carbohydrates presents a similar picture, though we noted inter-annual variation in carbohydrate levels (high in 2017) not seen in fatty acid levels. Finally, of the 1665 possible unique pairwise correlations between nutritive components in the full analyses, all are positive, the vast majority (1336, or 80.2%) significantly so.

## Conclusion

We conclude that Chl-*a* density measures provided by the chlorofluorometer provide a reliable and useful way to assess the abundance of biofilm in a manner relevant to foraging by western sandpipers.
